# Polydopamine Antioxidant Hydrogels for Wound Healing Applications

**DOI:** 10.3390/gels6040039

**Published:** 2020-10-31

**Authors:** Naphtali A. O’Connor, Abdulhaq Syed, Madeline Wong, Josiah Hicks, Greisly Nunez, Andrei Jitianu, Zach Siler, Marnie Peterson

**Affiliations:** 1 Department of Chemistry, Lehman College of the City University of New York, Bronx, NY 10468, USA; abdulhaq.syed@lc.cuny.edu (A.S.); madeline.wong@lc.cuny.edu (M.W.); Josiah.Hicks1@lehman.cuny.edu (J.H.); GREISLY.NUNEZ1@lehman.cuny.edu (G.N.); andrei.jitianu@lehman.cuny.edu (A.J.); 2 Ph.D. Programs in Chemistry and Biochemistry, The Graduate Center of the City University of New York, New York, NY 10016, USA; 3 Perfectus Biomed, LLC, Jackson Hole, WY 83001, USA; zach@perfectusbiomed.com (Z.S.); marnie@perfectusbiomed.com (M.P.)

**Keywords:** hydrogels, antioxidant, polydopamine, wound healing, biomaterials

## Abstract

Antioxidants are known to improve the wound healing process and are researched as a therapeutic strategy to treat chronic wounds. Dopamine is a known neurotransmitter with antioxidant properties that can be polymerized to form polydopamine (PDA). Herein, polydopamine is demonstrated as an antioxidant biomaterial. In prior work, we developed methodology to prepare hydrogels by crosslinking polysaccharides with polyamines via epichlorohydrin and NaOH. Using this previously developed methodology, dextran hydrogels crosslinked with polydopamine were prepared. Darkening of the gels indicated the increasing incorporation of polydopamine within the hydrogels. In addition to basic pH, polydopamine can be formed by reaction with polyethylene imine (PEI), which results in PEI-PDA copolymer. Dextran was similarly crosslinked with the PEI-PDA copolymer and resulted in sturdier, darker gels, which had more polydopamine incorporated. Hydrogel morphology and strength were dependent on the feed ratios of dopamine. Antioxidant activity of polydopamine containing hydrogel was confirmed and shown to be dependent on the amount of dopamine used in hydrogel synthesis. Hydrogels with 0.5 dopamine to dextran feed ratio scavenged 78.8% of radicals in a 2,2′-azinobis-(3-ethylbenzothiazoline-6-sulfonic acid) antioxidant assay while gels with no dopamine scavenged only 1.4% of radicals. An ex vivo wound healing assay showed considerable cell migration with the PEI-PDA containing hydrogel.

## 1. Introduction

Hydrogels are networked polymers that absorb a large amount of water. They are attractive for wound care as they provide a moist wound environment, debridement of the wound area, absorbance of excess exudate and low adherence to wounds [1]. Chronic wounds are difficult to heal wounds with an out of control inflammatory stage, characterized by high levels of oxidative stress, protease activity and infections [2,3]. Antioxidants such as vitamin E and flavonoids have been shown to accelerate the healing of cutaneous wounds and are widely researched as a therapy for chronic wounds [4]. One strategy to deliver antioxidants to wounds is to have them absorbed by hydrogels and released by diffusion [5,6]. With this strategy, the antioxidant activity will be dominated by the absorption and release properties of the hydrogel. Another strategy is to graft antioxidants to hydrogels [7,8,9]. This requires additional synthetic steps and functional groups in the hydrogel antioxidant to make synthesis possible.

Dopamine is a neurotransmitter with antioxidant activity from its catechol functional group [10]. It oxidizes in aqueous solution at basic pH to polymerize and form polydopamine. Polydopamine (PDA) is widely researched as a coating for biomaterials and is often used to mimic the adhesive proteins of mussels because of its numerous catechols [11,12,13]. These catechols also impart antioxidant activity to PDA [14]. Herein, we demonstrate that PDA can be used to make hydrogels into antioxidants. Polyethylene imine (PEI) and dopamine were recently shown to copolymerize to form a PEI-PDA conjugate in one pot [15]. This reaction is facilitated by the Michael addition of the PEI amine onto the 5,6-dihydroxyindole units of PDA. Recently, we reported the one-pot synthesis of hydrogels by crosslinking polysaccharide with polyamines such as PEI [16,17]. This is a two-step, one-pot process where polysaccharides are first reacted with epichlorohydrin. The reaction is biphasic and epichlorohydrin is considered consumed when the reaction becomes homogeneous. The polyamine is then added to the reaction mixture to prepare the hybrid gels. Herein, we have used this methodology to demonstrate the facile synthesis of antioxidant PDA-based hydrogels with antioxidant properties confirmed by a 2,2′-azinobis-(3-ethylbenzothiazoline-6-sulfonic acid) radical cation (ABTS•+) decolorization assay [18]. The PEI-PDA copolymer was crosslinked with dextran and this resulting hydrogel facilitated cell migration in an ex vivo wound healing study and indicated that polydopamine may be a material that warrants further interest for developing wound healing and tissue engineering applications.

## 2. Results and Discussion

### 2.1. Synthesis and Structure

We previously reported a one-pot process for the preparation of polysaccharide-polyamine crosslinked hydrogels and this process was adapted to produce dextran hydrogels crosslinked with polydopamine (PDA) and a PEI-PDA copolymer [16,17]. Dextran was reacted with epichlorohydrin for approximately 30 min until the biphasic mixture became homogeneous. This is taken as the point where all the epichlorohydrin has reacted with dextran. Polydopamine can be prepared from dopamine under basic conditions and thus dopamine was added to the basic dextran mixture, upon which the solution becomes brown, indicating the PDA formation. Dex-PDA (dextran-polydopamine) gels were prepared by varying the mass ratio from 2:1–2:0.2 dextran to dopamine with increased dopamine correlating with darker gels (Figure S1). Synthesis of these gels was noticeably sensitive to the order and timing of stirring. This sensitivity increased with increasing the proportion of dopamine included in the gel. This is most likely due to dopamine reacting with epichlorohydrin and reducing the crosslinking density necessary for gelation. Dex-PDA gels did not form within the first 7 h and were allowed to stand for 24 h, after which gels were observed.

**Figure 1:**
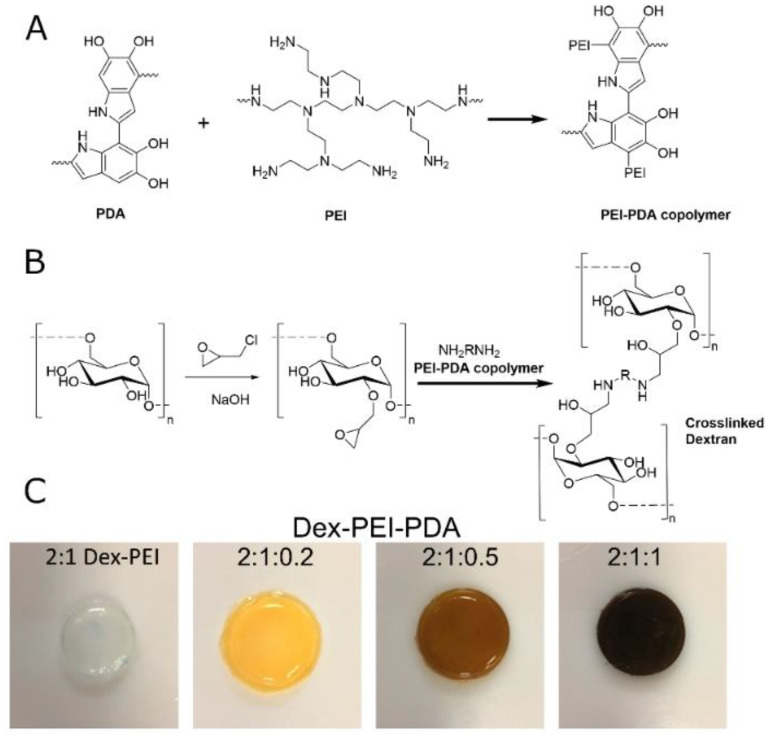
(**A**) Polyethylene imine-polydopamine (PEI-PDA) copolymer synthesis, (**B**) dex-PEI-PDA (dextran-polyethylene imine-polydopamine) hydrogel synthesis, (**C**) dex-PEI-PDA representative hydrogels with feed ratios of dextran:PEI:dopamine ranged 2:1:1–2:1:0.2.

PEI has previously been shown to react with dopamine to form PEI-PDA copolymers by the Michael addition of the PEI amine onto the 5,6-dihydroxyindole units of PDA [15]. To prepare dex-PEI-PDA (dextran-polyethylene imine-polydopamine) gels, PEI was first reacted with dopamine for up to 1 h and a brown coloration signified the formation of polydopamine. Separately, dextran was reacted with epichlorohydrin for approximately 30 min until homogeneous and then the PEI-PDA copolymer was added to crosslink the polysaccharide to the polyamine and the solution. Gels formed within 1 h and were allowed to stand for 24 h. Gelation was faster than the process that excluded PEI and the dex-PEI-PDA gels were darker than the dex-PDA gels, indicating greater incorporation of polydopamine. These gels were made with varying amounts of dopamine with mass ratios ranging 2:1:1–2:1:0.2 for dextran:PEI:dopamine (Figure 1).

**Figure 2:**
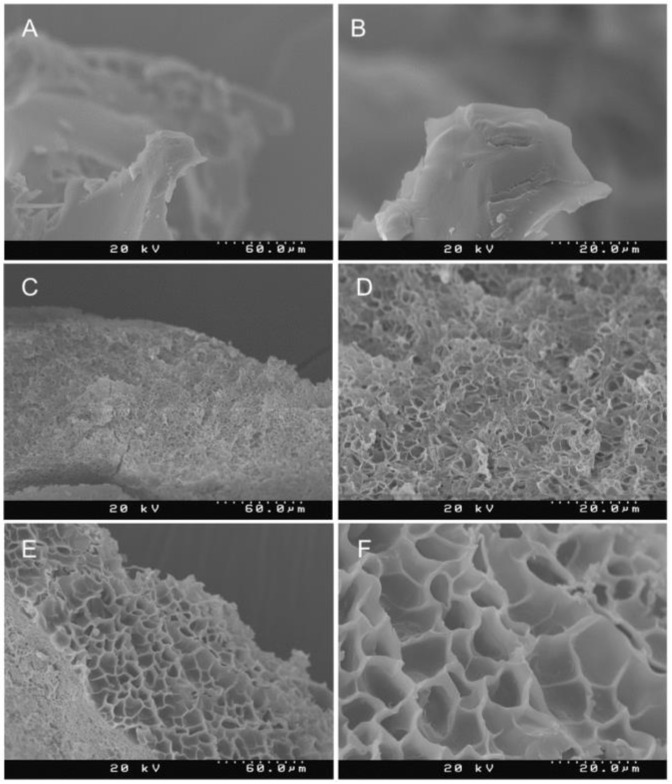
SEM images of hydrogels: (**A**) dextran at 500×, (**B**) dextran at 1500×, (**C**) dex-PDA with feed ratio of 2:1 at 500×, (**D**) dex-PDA with feed ratio of 2:1 at 1500×, (**E**) dex-PEI-PDA with feed ratio of 2:1:1 at 500×, (**F**) dex-PEI-PDA with feed ratio of 2:1:1 at 1500×.

SEM micrographs showed gel morphology to be dependent on gel composition (Figure 2). The hydrogel of only dextran (Figure 2A,B) showed untextured sheets lacking in porosity. Hierarchical pore structures were observed in the dex-PDA gel comprised of dextran and dopamine in a 2:1 mass ratio. The pores appeared to be approximately 2–4 μm in diameter. Sponge-like features with a high polydispersity of the pores were also observed for the 2:1:1 dex-PEI-PDA gels; however, the pore size of this sample was much larger in size, with diameters in the range of 5–15 μm.

**Figure 3:**
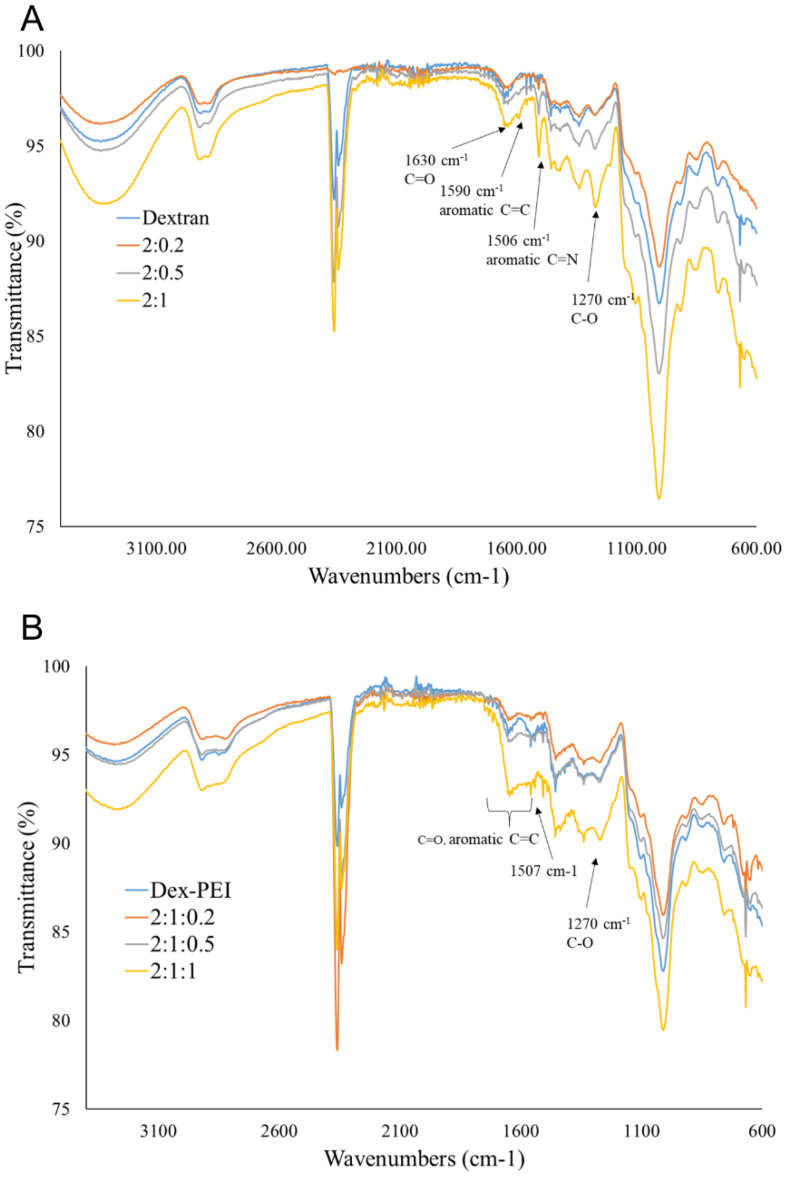
FTIR spectra of (**A**) dex-PDA with feed ratios ranging 2:1–2:0.2 (dextran hydrogel has no PDA present) and (**B**) dex-PEI-PDA with feed ratios ranging 2:1:1–2:1:0.2 (dex-PEI hydrogel has no PDA present).

Fourier-transform infrared (FTIR) spectroscopy confirmed the incorporation of both dextran and dopamine within the dex-PDA gels (Figure 3A). The characteristic polysaccharide OH and CH stretches were observed at 3300 and 2912 cm^−1^ [19]. Another peak associated with dextran is the intense peak at 1002 cm^−1^, which is likely due to chain flexibility of the α (1→6) glycosidic bond. As the proportion of dopamine increases in dex-PDA gels, notable increases are noticed at 1590 and 1506 cm^−1^, which were ascribed to aromatic C=C and C=N stretching modes [20]. Increases in the regions attributed to C=C and C=N stretching were also observed in spectra of dex-PEI-PDA gels, confirming the PDA within the gels. It was difficult to attribute peaks to PEI due to overlap with other absorptions (Figure 3B).

### 2.2. Swelling, Antioxidant and Compression Studies

**Figure 4:**
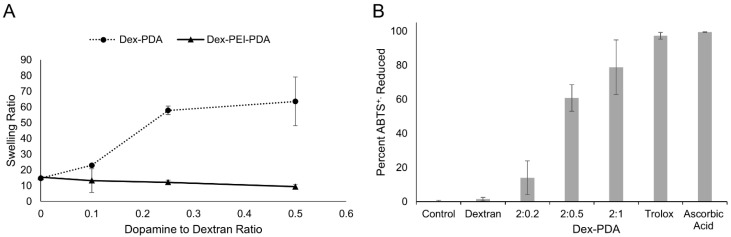
(**A**) Swelling ratios for dex-PDA hydrogels in deionized water with feed ratios ranging 2:1–2:0.2 (dextran hydrogel has no PDA present) and dex-PEI-PDA hydrogels with feed ratios ranging 2:1:1–2:1:0.2 (dex-PEI hydrogel has no PDA present). (**B**) The 2,2′-Azino-bis(3-ethylbenzothiazoline-6-sulfonic acid) diammonium salt (ABTS) antioxidant assay of dex-PDA hydrogels with feed ratios ranging 2:1–2:0.2. Negative controls of dextran hydrogel without PDA and water were used. For a positive control antioxidants Trolox and ascorbic acid were used.

An analysis of swelling reveals that the inclusion of dopamine in dex-PDA gels increases their swelling ratio in contrast to control gels of only dextran (Figure 4A). Average swelling in dextran only gel was 14.8 compared to 23.0 in dex-PDA gel with a dopamine to dextran feed ratio of 0.1 (2:0.2 dex-PDA). The swelling increases considerably to 57.9 when the dopamine to dextran feed ratio is increased further to 0.25 (2:0.5 dex-PDA). This appears to level off with gels with a feed ratio of 0.5 (2:1 dex-PDA), having an average swelling of 63.6 but being within the margin of error with gels with a feed ratio of 0.25. It is likely that polydopamine reduces the crosslinking by consuming epichlorohydrin. As such, increased swelling of dex-PDA gels when compared to the dextran only gels is to be expected. Little variation was observed in dex-PEI-PDA gels when compared to a control gel of dextran and PEI without PDA present. The PEI dominates the swelling and gelation behavior as its inclusion speeds up gelation regardless of the amount of dopamine included.

Dopamine is known to have antioxidant properties, thus the inclusion of PDA in gels is predicted to incorporate antioxidant activity in the gels. The antioxidant activity of dex-PDA gels was examined using the ABTS antioxidant assay where antioxidant activity is measured by the ability to scavenge ABTS radical cations (Figure 4B). Antioxidant activity was observed to be dependent on the amount of PDA within the gels. While the dextran only gel showed no antioxidant activity with 1.4% of the ABTS radical cations scavenged, the 2:0.2 dex-PDA gel had an average of 13.9%. When the ratio of PDA is increased further in the 2:0.5 and 2:1 dex-PDA gels, the percentage of ABTS radical cations scavenged increased to 60.7 and 78.8%, respectively. This confirms that PDA imparts antioxidant properties upon the gels and that the activity can be controlled by the amount of dopamine used in the formulation. PEI on its own decolorizes the ABTS assay and because of this the antioxidant activity of PDA within the dex-PEI-PDA gels was not confirmed.

**Figure 5:**
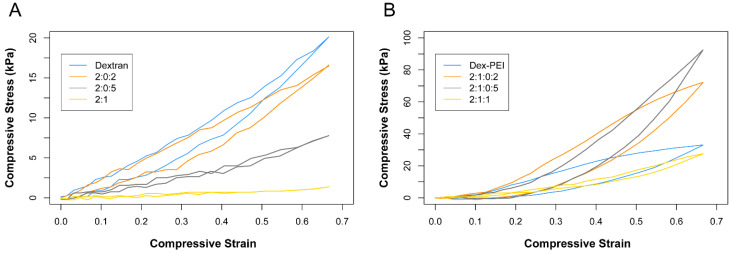
Compressive stress vs. compressive strain curves for (**A**) dex-PDA hydrogels with feed ratios ranging 2:1–2:0.2 (dextran hydrogel has no PDA present). (**B**) dex-PEI-PDA hydrogels with feed ratios ranging 2:1:1–2:1:0.2 (dex-PEI hydrogel has no PDA present).

Representative compressive stress vs. strain curves for dex-PDA and dex-PEI-PDA gels are shown in Figure 5. Dopamine reduces the stiffness and strength of dex-PDA gels with all formulations showing lower compressive stress for 65% compressive strain (Figure 5A). Dextran only gels required 20.1 kPa of compressive stress for 65% compressive strain and this reduced to 16.6 when the dopamine to dextran ratio was 0.1 (2:0.2 dex-PDA). Increasing the dopamine to dextran ratio to 0.25 (2:0.5 dex-PDA) saw this decrease further to 7.8 kPa. The 2:1 dex-PDA gels with the highest dopamine to dextran ratio of 0.5 required the least compressive stress of 1.3 kPa for 65% compressive strain. The decrease in strength and stiffness with increased dopamine correlates inversely with swelling ratio. This can be correlated with the porosity of the sample and with the presence of the hierarchical pore structures. A less crosslinked gel with a higher dopamine content can be expected to have lesser stiffness and strength.

The PEI-based gels showed considerably higher compressive stress at 65% compressive strain (Figure 5B). Dex-PEI gel with no dopamine required compressive stress of 33.1 kPa for 65% compressive strain. This increases to 72.2 and 92.5 kPa for gels with a dopamine to dextran ratio of 0.1 (2:1:0.2 dex-PEI-PDA) and 0.25 (2:1:0.5 dex-PEI-PDA), respectively. This trend is the opposite of what was seen in dex-PDA gels, as the dopamine increases, the strength and stiffness of the gels increase. From a porosity point of view, those samples present a wide pore size distribution. However, the walls of those pores might be more rigid due to the necessity of supporting the 3D structure, which will determine the higher stiffness of the sample. This trend ends when the dopamine to dextran ratio was 0.5 (2:1:1 dex-PEI-PDA) as the compressive stress fell to 29.9 kPa.

### 2.3. Wound Healing Studies

**Figure 6:**
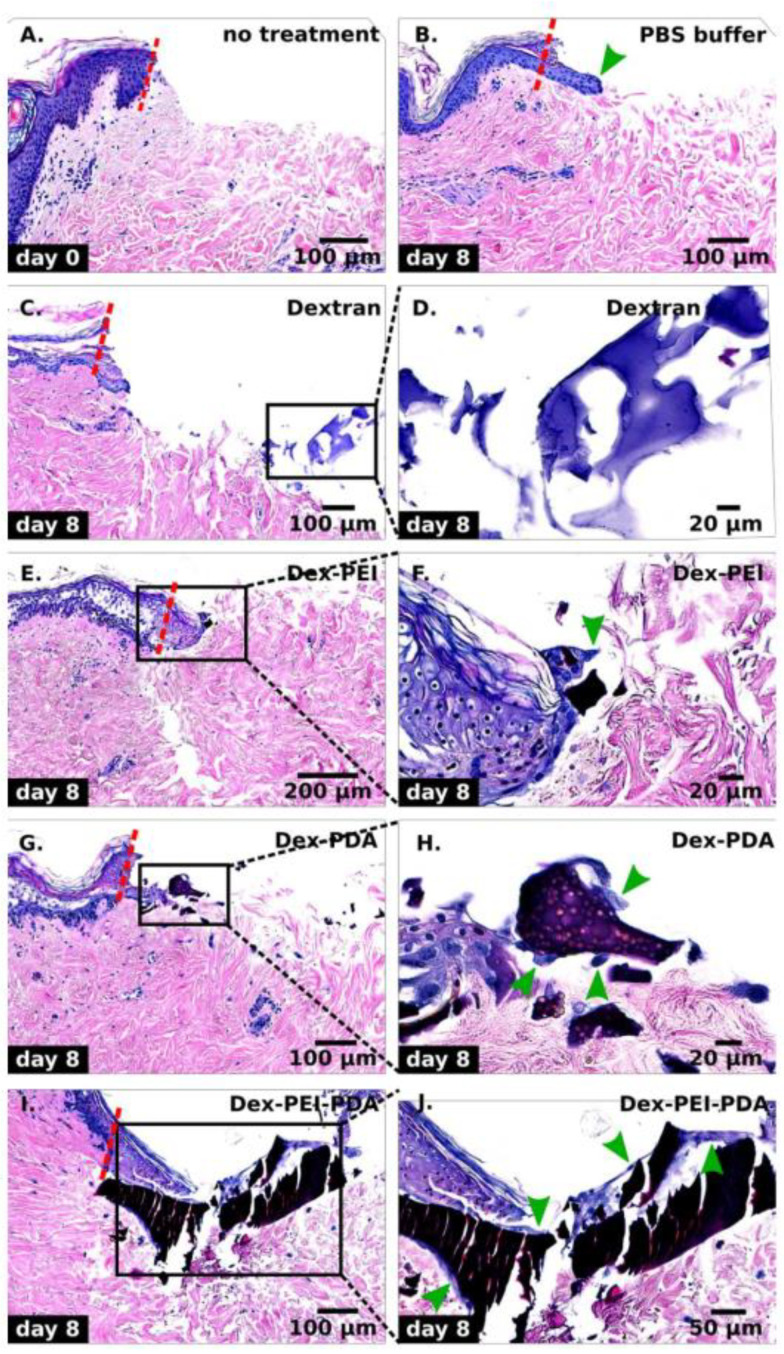
Hematoxylin & Eosin (H&E) stain of transverse section of human abdominal skin explants at 63× after 8 days of treatment with hydrogels. (**A**) Wound created on day 0. (**B**) Phosphate buffered saline (PBS) buffer. (**C**,**D**) Dextran hydrogel and magnified area. (**E**,**F**) 2:1 dex-PEI hydrogel and magnified area. (**G**,**H**) 2:1 dex-PDA hydrogel and magnified area. (**I**,**J**) 2:1:1 dex-PEI-PDA hydrogel and magnified area. Green arrows indicate epithelial cells interacting with the hydrogels.

An ex-vivo model was employed to examine the effect of polyamines on the wound healing process. Cellular wound healing assays were deemed not viable because polydopamine makes the gels dark and opaque (Figure 1C). This makes brightfield imaging difficult. Furthermore, polydopamine has broad absorption and fluorescence spectra, making fluorescent cellular imaging also difficult [15]. The gels also do not possess the stiffness required to sufficiently grow cells on their surface, which would be a requirement for a cellular wound healing assay [21]. Wounds were created on human abdominal explants, which were then treated with suspensions of ground hydrogels (Figure S2). Ground hydrogels were used because of the fragility of some of the hydrogels. The effect of hydrogels on re-epithelization is made by comparison to the control experiment with phosphate buffered saline (PBS) after eight days (Figure 6B). With PBS buffer, re-epithelization of a little over 100 μm was observed. Considerably less re-epithelization was observed with the dextran hydrogel (Figure 6C) and a magnification of residual gel is seen in Figure 6D. Re-epithelization improved compared to dextran with 2:1 dex-PEI and 2:1 dex-PDA gels (Figure 6E,G), but no improvement over the control/PBS buffer. An interesting phenomenon was observed in the magnified image of the wound treated with 2:1 dex-PDA gels in Figure 6H. Re-epithelization had entrapped some of the gel pieces and epithelial cells are observed attached to a large piece of gel. This effect is considerably more pronounced in the wound treated with 2:1:1 dex-PEI-PDA gels (Figure 6I,J), where more re-epithelization was observed. The re-epithelization occurred above and below the dex-PEI-PDA gel that was trapped in the wound bed. This indicates that polydopamine materials may have interesting properties regarding cell migration and re-epithelization. From our observations, dex-PEI-PDA gels contain considerably greater amounts of polydopamine than dex-PDA and is likely the reason for the difference observed between the two gels. In addition to having antioxidant properties, polydopamine is widely researched as a biomaterial for its adhesive properties, and this may be what has accelerated the cell migration so starkly compared to the other hydrogels. 

## 3. Conclusions

Polydopamine has been successfully demonstrated as a source for developing antioxidant biomaterials. It can be easily incorporated into biomaterials by tethering to amine functionalities. The swelling, antioxidant, strength and stiffness properties of the dex-PDA hydrogel series was directly related to the amount of dopamine used during synthesis. Swelling of the dex-PEI-PDA series is dominated by the high crosslinking of PEI with no significant changes with the inclusion of dopamine. However, the strength and stiffness varied with the dopamine content. Considerable cell migration was observed on the surface of the 2:1:1 dex-PEI-PDA gel, indicating that polydopamine may be a biomaterial of great value to wound healing and tissue engineering research. 

## 4. Materials and Methods

### 4.1. Materials

Dextran (MW 500 kDa) and dopamine hydrochloride were obtained from Alfa Aesar (Tewksbury, MA, USA). Epichlorohydrin was obtained from Acros Organics (Geel, Belgium). Polyethylene imine (M_r_ 600–1000 kDa) was obtained from Hampton Research (Aliso Viejo, CA, USA). The Trolox Equivalent Antioxidant Capacity (TEAC) Assay Kit was obtained from Fisher Scientific (Pittsburgh, PA, USA). Characterization with Attenuated Total Reflection (ATR)-FTIR was performed using a Nicolet iS10 FTIR (Thermo Fisher Scientific, Waltham, MA, USA) and mechanical testing was done with a Cellscale Univert (Waterloo, ON, Canada) uniaxial tension/compression tester. To visually examine the hydrogel microstructures, the surfaces were evaluated using a HITACHI S-2700 SEM (Santa Clara, CA, USA). The SEM was operated at 20 keV acceleration voltage. The SEM was equipped with an Advanced Microscopy Techniques (Woburn, MA, USA) digital camera system. The lyophilized hydrogel was mounted onto an aluminum stub using carbon tape and then sputtered with gold prior to analysis to avoid charging.

### 4.2. Synthesis of Dextran:Polydopamine (Dex-PDA) and Dextran:PEI:Polydopamine (Dex-PEI-PDA) Hydrogels

This method of hydrogel synthesis was detailed in our prior publication [16]. To a stirring solution consisting of dextran (20% *w/v*, 500 μL), sodium hydroxide (5 M, 200 μL), and epichlorohydrin (75 μL) that had been mixed until homogeneity, dopamine hydrochloride was added. The solutions were continuously mixed until a brown color denoting the formation of polydopamine developed. The solutions were then left to sit for 24 h to allow the gels to set. The gels were then washed with water to remove excess NaOH and dopamine products. The release of dopamine products was monitored by a Lambda 35 UV–VIS spectrometer (Perkin Elmer, Waltham, MA, USA). Dex-PDA hydrogels were produced from dextran:dopamine feed ratios ranging from 2:1–2:0.2. Dex-PEI-PDA gels were prepared similarly; however, PEI (20% *w/v*, 250 μL) and dopamine were mixed for 1 h before adding to the dextran solution. The dextran:PEI:dopamine feed ratio for these gels ranged from 2:1:1–2:1:0.2. The gels were washed similarly to the dex-PDA gels. The washings were tested with a ninhydrin stain to confirm that PEI release was nominal. As a control for several experiments described below, dextran (20% *w/v* 500 μL) was crosslinked similarly with sodium hydroxide (5 M, 200 μL) and epichlorohydrin (75 μL) without the subsequent addition of a polyamine.

### 4.3. Swelling Ratio

The gels were partitioned into quarters and immersed in 100 mL deionized water for 24 h to obtain the swollen weight. For the swollen weight, excess water was removed by placing the gels on a makeshift drain and gently patting with Kimwipes. Drying of the gels was done in a vacuum oven at 75 °C for 24 h to obtain their dried weight. The swelling ratio was calculated using the following equation: $\frac{w-w_{o}}{w_{o}}$, where *w* is the swollen weight and *w_o_* is the dried weight. The swelling ratio was determined by averaging 3 measurements and the error determined by their standard deviation. 

### 4.4. Compression Studies

Circular hydrogel specimens for testing were prepared using hole punchers with diameters of 10 mm. The specimens were preconditioned for 30 cycles at 10% strain with 5 s for compression and 5 s for recovery per cycle. Specimens were axially compressed to 65% strain with 5 s for compression and 5 s for recovery.

### 4.5. Antioxidant Studies

The antioxidant behavior of the gels was tested using the Trolox Equivalent Antioxidant Capacity (TEAC) Assay [18]. First, 2,2′-Azino-bis(3-ethylbenzothiazoline-6-sulfonic acid) diammonium salt (ABTS) was reacted with potassium persulfate 24 h before the experiment to create a stable ABTS radical cation. The solution was left in the dark for 12–16 h prior to use. The ABTS solution was diluted ten-fold for the experiment. Gel samples were dried and ground into a fine powder prior to the experiment. Then, 15 mg of each sample was incubated in the ABTS solution for 1 min and then centrifuged at 2000 rpm for 30 s. The solution was removed and the decolorization was obtained using the UV–VIS spectrometer between the wavelengths of 380–900 nm. Positive control antioxidant behavior for the studies was obtained using corresponding amounts of Trolox and ascorbic acid as a standard. The antioxidant activity was determined by averaging 3 measurements and the error determined by their standard deviation.

### 4.6. Wound Healing Studies

Perfectus Biomed, LLC has been granted an exemption by Solutions Institutional Review Board (IRB) of Yarnell, AZ (08 Jan 2019) under IRB protocol #2018/12/2 for use of human tissue in ex vivo models to test antimicrobial and wound-healing products. Deidentified human abdominal skin discarded from elective plastic surgery procedures was obtained under this IRB protocol. Full-thickness abdominal skin was placed on sterile saline gauze in a sealed container within 30 min post-surgery and transported to the laboratory. At the laboratory, skin was immediately placed in Dulbecco’s modified Eagle’s Medium (DMEM) and 2% penicillin/streptomycin and 10% fetal bovine serum and refrigerated at 20 °C for 30 min. Skin was then placed on a disinfected wax dissection tray, corners were pinned down epithelial side up, and skin surface was wiped with 70% EtOH. A 2-mm biopsy punch was used to make superficial wounds (<1 mm deep) on the surface of the skin, and then a 5-mm biopsy punch was used around the 2-mm wound to create full-thickness explants. Wounded skin explants (*n* = 2) were transferred wound side up onto semipermeable inserts resting in DMEM and 2% penicillin/streptomycin and 10% fetal bovine serum. Hydrogels for wound healing studies were freeze dried and ground into a powder and made into a slurry in sterilized water at a concentration of 500 mg of gel/10 mL. The hydrogels examined were dextran, 2:1 dex-PEI, 2:1 dex-PDA and 2:1:1 dex-PEI-PDA. PBS buffer was used as a control. Wounds were treated with 50 μL of the slurry formulation or 2-mm diameter discs of hydrogel formulations. Explants were then incubated at 37 °C (7% CO_2_, 65% humidity) for 0, 96, and 192 h. Each day, 5 μL of appropriate slurry formulation was added on top of the treatment area for hydration and growth medium was replenished in the wells. At collection timepoints, explants were transferred to 500 μL of formalin and transported to a local pathologist for tissue processing and H&E staining. Histology slides were visualized with an Olympus BX63 microscope at 63×.

## Supplementary Materials

The following are available online at https://www.mdpi.com/2310-2861/6/4/39/s1, Figure S1: Representative image of hydrogels with feed ratios of dextran:dopamine ranging 2:1–2:0.2, Figure S2: H&E stain of transverse section of human abdominal skin explants showing an untreated wound bed.
