# Supplementary material for: Polydopamine Antioxidant Hydrogels for Wound Healing Applications
Source: Gels. 2020 Oct 31;6(4):39. doi: 10.3390/gels6040039 (PMC7709666; doi:10.3390/gels6040039)
Supplement: Supplementary file 1 [file gels-06-00039-s001.pdf]

gels

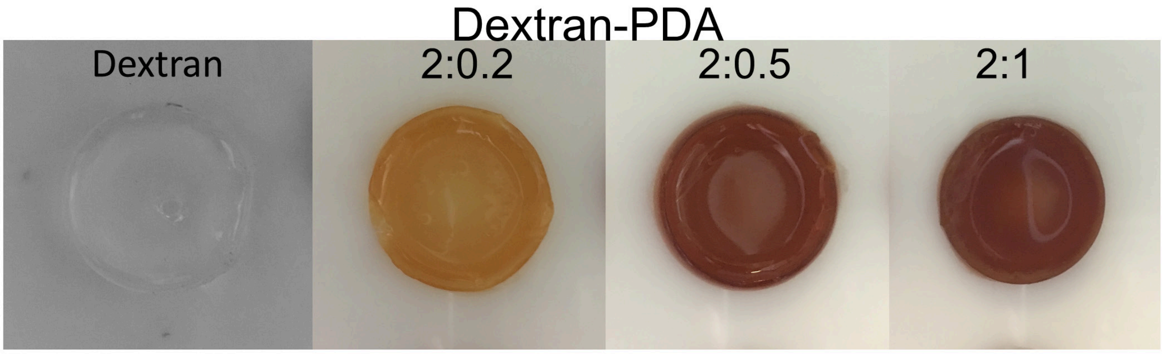

Figure S1. Representative image of hydrogels with feed ratios of dextran:dopamine ranging 2:1 – 2:0.2.

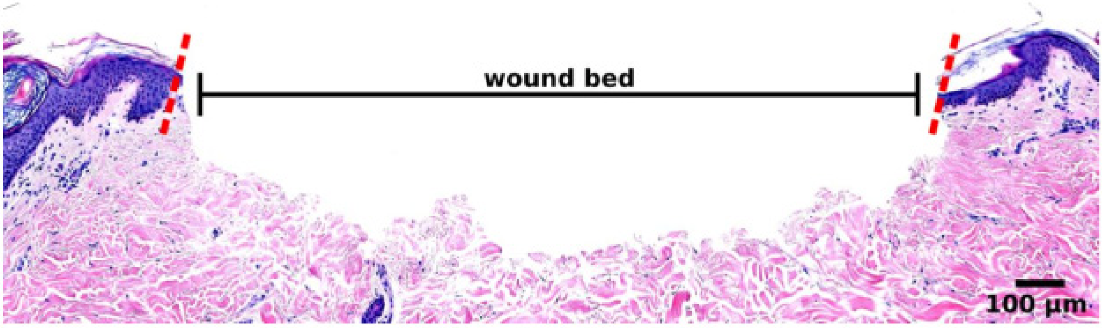

Figure S2. H&E stain of transverse section of human abdominal skin explants showing an untreated wound bed.
